# Stroke Survivors’ Interaction With Hand Rehabilitation Devices: Observational Study

**DOI:** 10.2196/54159

**Published:** 2024-06-26

**Authors:** Chioma Obinuchi Wodu, Gillian Sweeney, Milena Slachetka, Andrew Kerr

**Affiliations:** 1 Department of Biomedical Engineering University of Strathclyde Glasgow United Kingdom; 2 Department of Biomedical Technology University of Port Harcourt Port Harcourt Nigeria

**Keywords:** stroke, rehabilitation, hand rehabilitation devices, accessibility, stroke survivors, rehabilitation technologies

## Abstract

**Background:**

The hand is crucial for carrying out activities of daily living as well as social interaction. Functional use of the upper limb is affected in up to 55% to 75% of stroke survivors 3 to 6 months after stroke. Rehabilitation can help restore function, and several rehabilitation devices have been designed to improve hand function. However, access to these devices is compromised in people with more severe loss of function.

**Objective:**

In this study, we aimed to observe stroke survivors with poor hand function interacting with a range of commonly used hand rehabilitation devices.

**Methods:**

Participants were engaged in an 8-week rehabilitation intervention at a technology-enriched rehabilitation gym. The participants spent 50-60 minutes of the 2-hour session in the upper limb section at least twice a week. Each participant communicated their rehabilitation goals, and an Action Research Arm Test (ARAT) was used to measure and categorize hand function as poor (scores of 0-9), moderate (scores of 10-56), or good (score of 57). Participants were observed during their interactions with 3 hand-based rehabilitation devices that focused on hand rehabilitation: the GripAble, NeuroBall, and Semi-Circular Peg Board. Observations of device interactions were recorded for each session.

**Results:**

A total of 29 participants were included in this study, of whom 10 (34%) had poor hand function, 17 (59%) had moderate hand function, and 2 (7%) had good hand function. There were no differences in the age and years after stroke among participants with poor hand function and those with moderate (*P*=.06 and *P*=.09, respectively) and good (*P*=.37 and *P*=.99, respectively) hand function. Regarding the ability of the 10 participants with poor hand function to interact with the 3 hand-based rehabilitation devices, 2 (20%) participants with an ARAT score greater than 0 were able to interact with the devices, whereas the other 8 (80%) who had an ARAT score of 0 could not. Their inability to interact with these devices was clinically examined, and the reason was determined to be a result of either the presence of (1) muscle tone or stiffness or (2) muscle weakness.

**Conclusions:**

Not all stroke survivors with impairments in their hands can make use of currently available rehabilitation technologies. Those with an ARAT score of 0 cannot actively interact with hand rehabilitation devices, as they cannot carry out the hand movement necessary for such interaction. The design of devices for hand rehabilitation should consider the accessibility needs of those with poor hand function.

## Introduction

Stroke is a major cause of disability in the world [1]. Globally, about 17 million people have a stroke each year [2]. In the United Kingdom, the prevalence of stroke is projected to rise from 950,200 to 2,119,400 cases between 2015 and 2035 [3]. This projected rise in the prevalence of stroke has been associated with improvements in medical advances that have led to a decline in the number of deaths due to acute stroke, among other reasons [4]. Nevertheless, stroke survivors are faced with considerable long-term periods of enduring physical impairments, the likelihood of reoccurrence of strokes, transient ischemic attacks, or even death within 1 year of having a stroke [5]. Motor impairment (muscle weakness and the loss of movement control) is the most common consequence of stroke, impacting several aspects of life and reducing the ability of stroke survivors to lead an independent life [6]. About 55% to 75% of those who survive a stroke experience motor impairment in the upper limb 3 to 6 months after stroke [7].

The hand is crucial for carrying out activities of daily living such as eating, dressing, bathing, and communicating [8]. Besides, the hand is a defining feature of human beings and is vital for human daily interaction [9]. Due to this importance, impairments such as spasticity and weakness, which are common sequelae of stroke [10] and manifest in a fixed flexed position of the wrist and fingers, affect the function of the hand and impact the quality of life [10].

Rehabilitation can have a positive impact on the recovery of functions in persons with stroke [11] as well as in enhancing their quality of life [12], and movement restoration is a key goal in the rehabilitation of persons with neurological disorders [13]. The relearning of movement ability during rehabilitation is based on factors such as the repetitiveness, intensity, and regularity of task-specific movements [14]. It has been suggested that the rehabilitation of hand mobility and strength be prioritized once the general physical situation of stroke survivors has been stabilized owing to the importance of the hand [15].

**Figure 1:**
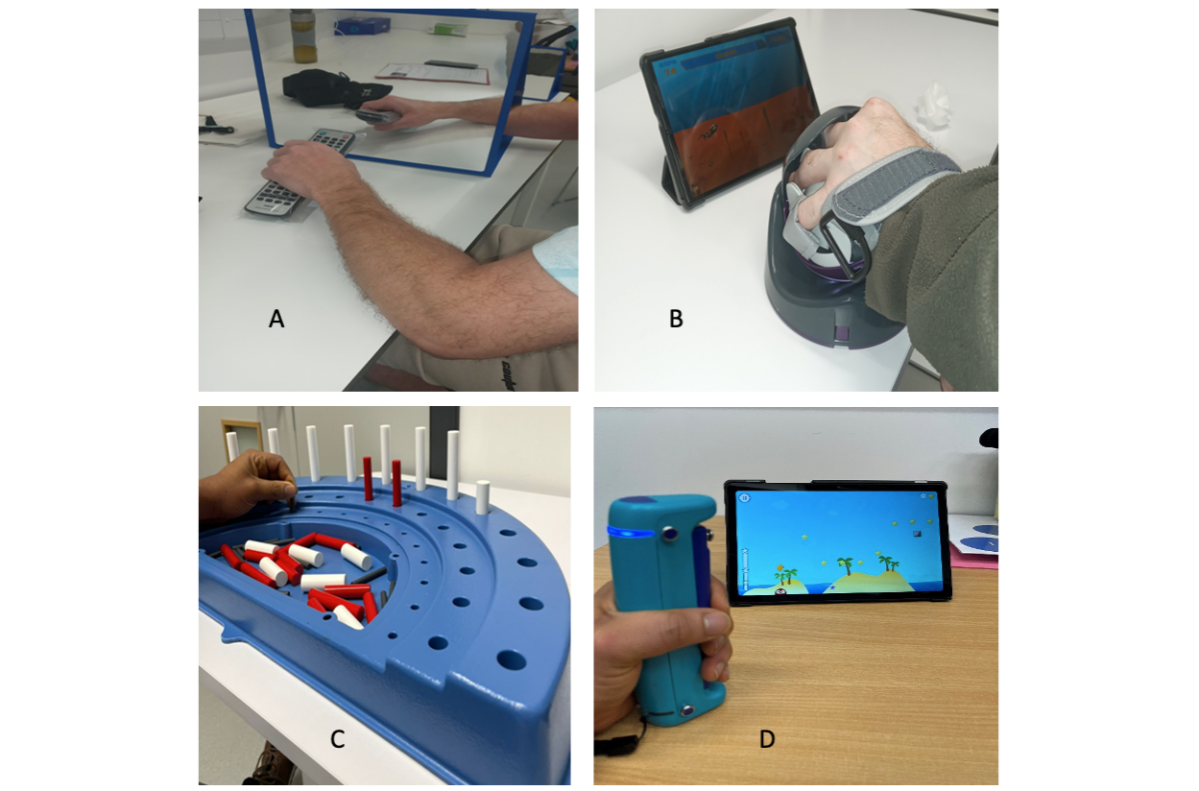
Upper limb rehabilitation technologies and tools used: (A) mirror (mirror therapy), (B) NeuroBall device, (C) Semi-Circular Peg Board, and (D) GripAble device.

Several new rehabilitation technologies that target the upper limb to improve motor functions are currently in use; these include the use of robotic-assisted technologies, virtual reality, and telerehabilitation [16]. Some others that are used in this study are gaming devices such as the GripAble (Gripable), NeuroBall (Neurofenix), and Semi-Circular Peg Board (Rolyan). The NeuroBall is an interactive device that connects wirelessly with a tablet app to carry out activities that can also be objectively measured [17]. The GripAble is a similar lightweight electronic handgrip [18] that also interacts wirelessly with a computer tablet, enabling users to interact with therapy games tailored to improve the upper limb and hand function in a way that can be objectively assessed [18,19]. The Rolyan Semi-Circular Peg Board consists of 3 colored pegs (red, white, and blue) of different diameters that the users are expected to pick up and place in their different peg holes (based on their diameter; see Figure 1 below). The ability of stroke survivors with poor hand function to access these devices is a major concern, as according to a report [20], only hemiplegic stroke survivors who are mildly disabled are likely to access hand or arm training apps that are available on mobile devices.

This study aims to observe stroke survivors’ interaction with hand rehabilitation devices and to understand how the different categories of hand function (Action Research Arm Test [ARAT] scores) influence the stroke survivors’ rehabilitation goals.

## Methods

### Participants

Participants were recruited from cohorts of stroke survivors attending a rehabilitation intervention at a cocreation center for accessible rehabilitation technology [21] between September 2021 and April 2023. The inclusion criteria for this study have been described in detail previously [21]; briefly, participants had to have had a stroke within the last 12 months that resulted in mobility problems, be aged over 18 years, be well enough to engage in light to moderate exercise, and be able to attend the rehabilitation program at least twice a week. A range of outcome measures were taken before and after the program, including the ARAT. An overview of the full rehabilitation program is available in our previously published report [21].

Out of a total of 36 participants who agreed to take part in the intervention, 7 (19%) were excluded from this study. Of the 7 excluded persons, 5 (71%) withdrew from the intervention (2/5, 40% withdrew before the commencement and 3/5, 60% withdrew due to ill health or unwillingness to continue), and the other 2 (29%) of the 7 were excluded as a result of incomplete data.

### The Upper Limb Rehabilitation Intervention

The upper limb intervention involved activities designed to improve the upper limb functions of participants, delivered completely through the use of technology and therapy devices that either stimulated or promoted repetitive and intensive movement training. The upper limb and hand rehabilitation technologies available to the participants in this study are shown in Table 1. The participants spent at least 50-60 minutes of each of the 2-hour sessions engaging with these devices.

**Table 1:** Upper limb rehabilitation technologies used.

Technology or device	Manufacturer	Function
GripAble	Gripable	It connects wirelessly with an app on a computer tablet [19] to interact with specifically designed therapy games [22], to train 4 different types of upper limb movements, such as grip and release, pronation and supination, wrist flexion and extension, and radius and ulnar deviations.
NeuroBall	Neurofenix	It connects wirelessly with a tablet app and interacts with therapy games specifically designed to exercise the upper limb of stroke survivors [17]. It trains upper limb movements such as finger grip; hand grip; right, left, upward, and downward tilt; and elbow and shoulder movements.
Mirror box	Saebo	It is a form of mental practice that excites the primary motor cortex, thereby evoking the movement of the affected limb, as the participants move the unaffected side while looking into the mirror [23].
Sensory TENS^a^	Med-Fit	It is a noninvasive nerve stimulator used to relieve pain [24], stimulate the muscles, and relieve muscle stiffness [25].
Semi-Circular Peg Board	Rolyan	It is a therapy tool designed to improve upper limb strength, movement coordination, endurance, and range of motion. It aims to improve hand dexterity.
Armeo Spring	Hocoma	It provides arm weight support while encouraging users to carry out self-initiated arm movements in the shoulder, elbow, and wrist joints and trains different upper limb movements [26].
Vibrating or hot compress massage ball	Dongguan Kooeej	It stimulates the hand using the vibrations delivered at different intensities.
VR^b^ headset	Occulus Quest with Incisiv software	It immerses the user into a virtual environment, thereby encouraging them to use their affected limb to interact with functional tasks [27,28].

^a^TENS: transcutaneous electrical nerve stimulation.

^b^VR: virtual reality.

### Overview of the Upper Limb Rehabilitation Program

**Figure 2:**
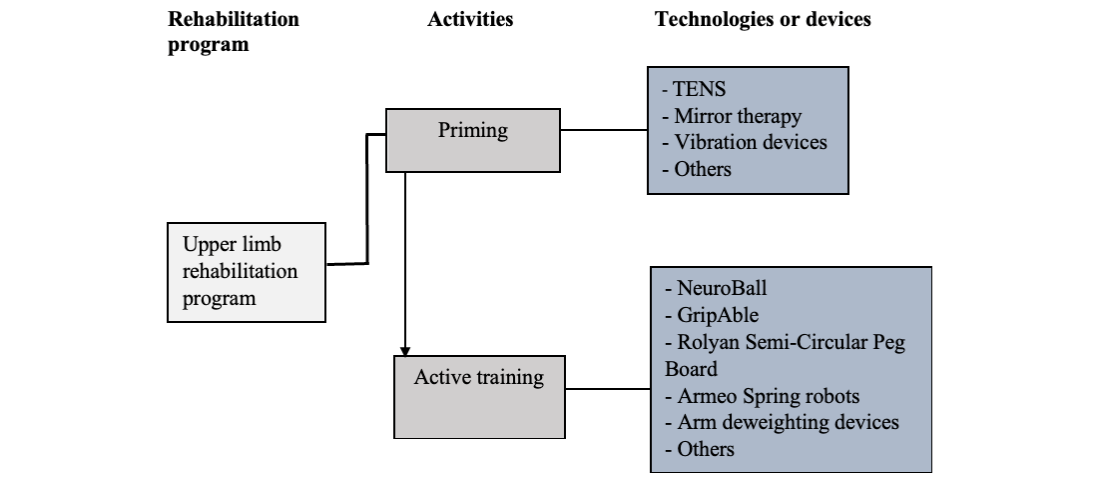
Upper limb rehabilitation program model for stroke survivors. TENS: transcutaneous electrical nerve stimulation.

Figure 2 is a representation of the upper limb rehabilitation program used in the rehabilitation gym. The activities were divided into 2 categories. The first part aimed at priming the brain to prepare it for plastic response [29]. Priming focused on sensory stimulation including mirror therapy and electrical, thermal, and vibrational stimulation. These priming activities comprised the first 15-20 minutes of each rehabilitation session. This second part, that is, the “active training,” aimed to engage the participants in high-intensity motor tasks such as object grip and release, object manipulation, and reach to grasp, designed to improve range of motion, strength, and control. The participants were not limited in terms of the number of devices they could use.

### Categorizing Participants Into Different Hand Function Groups

Participants were given a 1-day initial appointment with a therapist at the rehabilitation gym before the commencement of the 8-week rehabilitation intervention. During this appointment, demographic data including stroke history were collected, along with a range of baseline assessments for mobility, communication, and cognition, including the ARAT [30]. The ARAT was used to categorize the participants into 3 different hand function groups: poor (scores of 0-9), moderate (scores of 10-56), and good (score of 57) [30].

### Understanding the Rehabilitation Goals of Those With Different Categories of Hand Function

During the preintervention visit, participants were allowed to communicate their rehabilitation goals and interact with the upper limb devices to understand how they are set up and operated. The rehabilitation goals of the participants were summarized based on their different hand functions to help understand the needs of stroke survivors who fall under each of the different hand functions, particularly the hand rehabilitation goals of those with poor hand function.

### Observing the Interaction of Those With Poor Hand Function and the Hand Rehabilitation Devices

Following the goal setting and initial interaction with the devices, a rehabilitation program was drawn up. The rehabilitation program was individually tailored by a physiotherapist using the rehabilitation goals of the participants. The program however only acted as a guide, as participants had the freedom to interact with any of the devices. The ability of the participants to use each rehabilitation device was observed and recorded. At the end of the intervention, all the observations from participants with poor hand function were gathered and studied to see how they interacted with the hand-based rehabilitation devices. Three of the upper limb devices—the GripAble, NeuroBall, and Semi-Circular Peg Board (see Figure 1)—were selected for observation in this study. The reason for selecting these devices is because these 3 devices were the only devices listed under the “active training” category (see Figure 2) at the time of the study that were used to primarily train motor activities in the hand (involving the wrist and fingers) in addition to training other parts of the upper limb.

### Data Organization and Analysis

The simple percentage method was used to estimate the percentage of stroke survivors who fall into each category of hand function. A 1-way ANOVA was carried out using Minitab statistical software (Minitab LLC), with the Dunnett multiple comparison method used to compare the ages of the group with poor hand function to those with moderate and good hand function.

### Ethical Considerations

This study was approved by the University of Strathclyde ethics committee (approval UEC 20/08). The participants provided written informed consent before the study, and their participation was voluntary (no compensation was provided). All identifiable data were pseudoanonymized and replaced with a code.

## Results

### Categorizing Participants Into Different Hand Function Groups

Observations from 29 participants were included in this study. Their average age was 59.10 (SD 13.62) years with an average of 3.140 (SD 2.31) years after stroke. Of the 29 participants, 17 (59%) were hemiplegic on the left side of their body, whereas the remaining 12 (41%) were hemiplegic on the right side of their body (Table 2).

Of the 29 participants, 10 (34%) scored between 0 and 9 on the ARAT and were grouped as having poor hand function, 17 (59%) scored between 10 and 56 on the ARAT and were grouped as having moderate hand function, and 2 (7%) scored 57 on the ARAT and were grouped as having a good hand function. There was no statistical difference in age between the poor hand function group and both the moderate hand function (*P*=.06) and the good hand function (*P*=.37) groups. Similarly, there was equally no difference in the years after a stroke between the poor hand function group and both the moderate hand function (*P*=.09), and the good hand function (*P*=.99) groups. There was also no observed difference in the hemiplegic side of those with poor hand function (left: 5/10, 50%; right: 5/10, 50%).

**Table 2:** Characteristics of participants and the 3 subgroups.

Group	Participants (n=29), n	Hand function	Age (years), mean (SD)	Years after stroke, mean (SD)	Hemiplegic side, n (%)		ARAT^a^ score, mean (SD)
					Left	Right	
All	29 (100)	—^b^	59.10 (13.62)	3.14 (2.31)	17 (59)^c^	12 (41)^c^	26.63 (21.51)
1	10 (34)	Poor	64.70 (8.83)	2.10 (1.45)	5 (50)^d^	5 (50)^d^	2.00 (3.74)
2	17 (59)	Moderate	53.76 (13.89)	3.88 (2.57)	11 (65)^e^	6 (35)^e^	34.65 (16.09)
3	2 (7)	Good	76.50 (0.707)	2.00 (1.42)	1 (50)^f^	1 (50)^f^	57.00 (0.00)

^a^ARAT: Action Research Arm Test.

^b^Not applicable.

^c^n=29.

^d^n=10.

^e^n=17.

^f^n=2.

### Understanding the Rehabilitation Goals of Those With Different Categories of Hand Function

Table 3 shows a summary of the rehabilitation goals of stroke survivors based on their different hand functions. Participants with poor hand function stated goals that were more toward gaining movements in different parts of their upper limb, as well as improving the ability to carry out active movements that will enable them to grasp and release objects. However, stroke survivors with moderate and good hand function had goals that were focused on how to improve grip strength, fine motor movements, release time, as well as purposeful movement of the upper limb (see Table 3). Those with poor hand function who recorded a score greater than 0 on the ARAT equally communicated the need to improve grip strength.

**Table 3:** Upper limb and hand rehabilitation goals of participants separated into the 3 functional categories.

Group	Hand function	Rehabilitation goals as stated by the participants
1	Poor	Gain the ability to hold objects (eg, paper) Gain some shoulder movement Gain arm movement Recovery of any movement, primarily in the shoulder Improve the grasp and release of objects Improve active movements Grip strength^a^
2	Moderate	Improve dexterity Improve grip Improve the range of upper limb movement Improve upper limb strength Improve supination or pronation range Improve the grasp and release of objects Improve release time Gain the ability for small object manipulation Gain the ability to move objects Gain the ability for purposeful movement of the upper limb
3	Good	Increase grip Improve wrist extension

^a^For those who recorded a score >0 on the Action Research Arm Test (ARAT).

### Interaction With Hand Rehabilitation Technologies by the Poor Hand Function Group

Table 4 shows that 8 (80%) of the 10 participants with poor hand function could not interact with any of the 3 aforementioned devices to carry out active training. This value represents 28% (8/29) of the total population in this study. Only 2 (20%) of the 10 participants with poor hand function were able to engage with these devices; the ARAT score shows that these 2 participants had ARAT scores of 7 and 9, compared to the score of 0 that was recorded by the other 8 who were not able to engage with these devices.

**Table 4:** Interaction of stroke survivors who had poor hand function with the hand rehabilitation devices.

Participant ID	ARAT^a^ score	Upper limb rehabilitation goal	Use of devices for active hand training				Comments on the participants’ ability to use the devices
			GripAble	NeuroBall	Semi-Circular Peg Board		
1	0	General upper limb function	X^b^	X	X	Tightness in the hand and other parts of the upper limb did not allow the fitting of the devices into the hand	
2	0	Improve active movements	X	X	X	Weakness of the upper limb and hand; not able to carry out the active movement necessary for device usage	
3	0	Hold objects (eg, paper), gain some shoulder movement	X	X	X	Could not make use of any of the devices	
4	0	Improve the grasp and release of object	X	X	X	Difficult to initiate movement on the GripAble and NeuroBall; could also not use the Semi-Circular Peg Board as a result of weakness in the hand	
5	7	Grip strength, range of shoulder or elbow active movement	✓^c^	✓	✓	Fought to maintain grip due to the presence of tightness; the participant noted that “Botox [had] not helped a lot” with hand function. However, they were able to make use of the devices	
6	0	Gain arm movement	X	X	X	Upper limb and hand stiffness affected the ability to access the devices	
7	0	Would like to get some movement	X	X	X	Had very limited movements	
8	0	Recovery of any movement, primarily in the shoulder	X	X	X	Weakness of the upper limb and hand; not able to carry out active movement necessary for device usage	
9	0	—^d^	X	X	X	Attempted the GripAble and NeuroBall once but was not able to make use of them	
10	9	Grip strength	✓	✓	✓	—	

^a^ARAT: Action Research Arm Test.

^b^X: unable.

^c^✓: able.

^d^Not applicable.

## Discussions

### Principal Findings

This study was carried out to observe how stroke survivors with poor hand function interacted with hand rehabilitation devices such as the GripAble, NeuroBall, and Semi-Circular Peg Board. The findings show that stroke survivors whose poor hand function leads to an ARAT score of 0 cannot actively interact with hand rehabilitation devices.

### Comparison to Prior Work

About two-thirds (55%-75%) of persons who had a stroke sustain upper limb impairments [7]. The extent of the impairments varies from person to person (see Table 2). In some, it results in poor hand function, whereas others present moderate or good hand function. The level of hand function present after stroke subsequently influences the upper limb rehabilitation goals of the stroke survivor (see Table 3). Stroke survivors with moderate to good hand function, who are likely to possess some range of motion in the hand, can grip, grasp, or pinch [30,31] hand rehabilitation devices and so have upper limb rehabilitation goals aimed at strengthening the existing motor ability. These goals may be related to improving grip strength and endurance, the ability to release objects or release time, the existing range of upper limb movements, and finger dexterity and regaining the ability to manipulate small objects (see Table 3). However, those with poor hand function, especially those with an ARAT score of 0 who cannot grasp, grip, or pinch objects irrespective of the sizes [31], have upper limb rehabilitation goals that focus on recovering some movement in the joints (shoulder, elbow, wrist, and/or fingers; see Tables 3 and 4).

**Figure 3:**
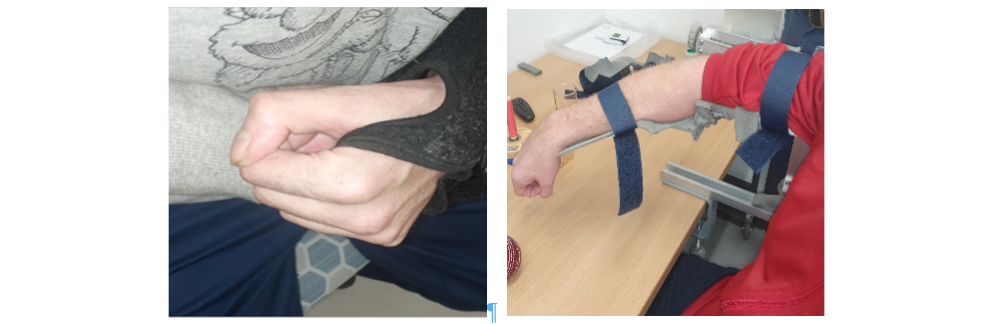
Participants with poor hand function taking part in the 8-week rehabilitation exercise.

Muscle weakness and the appearance of muscle stiffness, tightness, or tone (evident by the presence of a clenched hand) were clinically examined as being responsible for the poor hand function of the participants in this study (see Figure 3). The appearance of clenched hands has been reported as a clinical feature of spasticity [32]; moreover, the presence of muscle stiffness, tightness, and tone have all been connected with spasticity [33,34]. Previous studies have reported both spasticity and muscle weakness as the 2 major motor impairments following a stroke [35,36]. The severity of these impairments led to difficulty in hand immobility in 80% of those with poor hand function (with an ARAT score of 0), and according to an earlier report [36], spasticity and muscle weakness can result in immobility.

### Strengths

The *UK National Clinical Guideline for Stroke* stipulates that stroke survivors should be considered for rehabilitation at any point after the stroke to potentially gain benefits [37]. However, an earlier study [38] that measured the accuracy of physical therapists’ early prediction of upper limb function reported that stroke survivors with ARAT scores more than 10 are those principally qualified to undergo rehabilitation exercises; this potentially excludes stroke survivors with poor hand function from taking part in hand rehabilitation. This study shows that not all stroke survivors with poor hand function should be considered ineligible to make use of hand rehabilitation devices, as those with some range of motion in their hand, as seen in participants with ARAT scores of 7 and 9 (see Table 4), can still benefit from hand rehabilitation devices and thus active hand rehabilitation.

### Limitations

Only participants who exhibited poor hand function with an ARAT score of 0 were not able to benefit from active hand rehabilitation using devices. Those in this category whose poor hand function was due to muscle weakness were unable to carry out any intended active movement on the hand rehabilitation devices (see Table 4), even when supported to place their hand on them. In contrast, those whose poor hand function was due to hand stiffness or tightness, in addition to their inability to carry out intended active movement, were also faced with the problem of accessibility, which made it difficult for them to fit the device.

A limitation of this study was the inability to assess these conditions (muscle weakness and muscle tone or tightness)—examined to be responsible for the poor hand function—using the relevant outcome measures, such as motricity index, grip strength or pinch strength (for muscle weakness), or the Modified Ashworth Scale (for spasticity) [39], to quantify their severity. However, their severity was such that the hand was not useful in carrying out any of the ARAT tasks [31], as indicated by an ARAT score of 0.

### Future Direction

Improvement in technological advancement has led to the development of devices such as rehabilitation gloves (smart or robotic gloves) that can be useful in stretching the hands of stroke survivors with poor hand function without requiring their active participation [40,41]. However, only stroke survivors with low spasticity (who possess some range of active motion in the hand [42]) may be able to make use of these rehabilitation gloves [40]. This means those with considerable muscle stiffness resulting in difficulty in passive motion [42] are still unlikely to freely access these devices; thus, future design of rehabilitation devices for hand rehabilitation should consider the problem of device accessibility in people with poor hand function due to considerable muscle stiffness or tightness.

### Conclusions

It is therefore concluded that not all stroke survivors with impairments in their hands can interact with the available hand rehabilitation technologies, as those with an ARAT score of 0 cannot actively interact with any hand rehabilitation device. Thus, the selection of devices for hand rehabilitation should first consider the hand function of the affected stroke survivor. Since muscle stiffness or tightness in the hand results in poor hand function that can impede access to hand rehabilitation devices, future design of devices for hand rehabilitation should consider the accessibility needs of those with poor hand function as a result of hand stiffness or tightness. A similar observational study involving more stroke survivors will help ascertain the percentage of stroke survivors who fall into the category of having poor hand function and is therefore recommended.

## Abbreviations

ARAT: Action Research Arm Test

## Data Availability

The data sets generated during this study are available from the corresponding author upon reasonable request.
